# Brain volumes in fetuses with congenital heart disease and placental vascular abnormalities

**DOI:** 10.1038/s41372-026-02601-4

**Published:** 2026-03-13

**Authors:** Erin A. O’Brien, David Wypij, Valerie Rofeberg, Reem Chamseddine, Clemente Velasco-Annis, Kaylin Taylor, Kaysi Herrera Pujols, Nicholas J. Hart, Sarah U. Morton, Joshua Litwin, Nankee Kumar, Karem Kennedy, Louise Wilkins-Haug, Jane W. Newburger, Anthony O. Odibo, Ali Gholipour, Joshua S. Shimony, Caitlin K. Rollins, Cynthia M. Ortinau

**Affiliations:** 1 Department of Pediatrics, Vanderbilt University Medical Center, Nashville, TN USA; 2 Department of Cardiology, Boston Children’s Hospital, Boston, MA USA; 3 Department of Pediatrics, Harvard Medical School, Boston, MA USA; 4 Department of Biostatistics, Harvard T.H. Chan School of Public Health, Boston, MA USA; 5 Department of Neurology, Boston Children’s Hospital, Boston, MA USA; 6 Department of Obstetrics and Gynecology, The Brooklyn Hospital Center, Brooklyn, NY USA; 7 Department of Radiology, Boston Children’s Hospital, Boston, MA USA; 8 Department of Neurology, Washington University in St. Louis School of Medicine, St. Louis, MO USA; 9 Division of Newborn Medicine, Department of Pediatrics, Boston Children’s Hospital, Boston, MA USA; 10 St. Louis Children’s Hospital, St. Louis, MO USA; 11 Department of Obstetrics and Gynecology, Division of Maternal Fetal Medicine, Harvard Medical School, Brigham and Women’s Hospital in Boston, Boston, MA USA; 12 Department of Obstetrics and Gynecology, University Health Truman Medical Center, Kansas City, MO USA; 13 Department of Radiological Sciences, University of California Irvine, Irvine, CA USA; 14 Department of Electrical Engineering and Computer Science, University of California Irvine, Irvine, CA USA; 15 Mallinkrodt Institute of Radiology, Washington University in St. Louis School of Medicine, St. Louis, MO USA; 16 Department of Neurology, Harvard Medical School, Boston, MA USA; 17 Department of Pediatrics, Washington University in St. Louis School of Medicine, St. Louis, MO USA

**Keywords:** Developmental neurogenesis, Genetic testing, Congenital heart defects

## Abstract

**Objective:**

Investigate the association between placental vascular abnormalities and regional brain volumes in congenital heart disease (CHD) fetuses with and without genetic abnormalities.

**Study design:**

Secondary analysis of brain magnetic resonance imaging (MRI) and placental pathology data from 121 CHD fetuses enrolled in prospective neuroimaging studies at two centers.

**Results:**

Placental vascular abnormality was present in 46% of fetuses, and genetic abnormality was present in 19%, including 12% with both abnormalities. Fetuses with the combination of placental and genetic abnormalities had smaller brain volumes compared to fetuses without either abnormality for total brain, subcortical gray matter, brainstem, and cerebellum, with a significant interaction (*P* < 0.05) between placental and genetic abnormalities for intracranial and subcortical gray matter volumes.

**Conclusion:**

Smaller brain volumes for CHD fetuses with placental and genetic abnormalities may suggest common genetic pathways affect placental, heart, and brain development, or that genetic abnormalities heighten vulnerability when placental changes occur.

## Introduction

Survival of neonates with congenital heart disease (CHD) has dramatically improved over the last four decades, yet the neurodevelopmental (ND) morbidity of this population remains high [1]. A number of sociodemographic and clinical factors have been associated with adverse ND outcomes in CHD [1, 2]. Recent data accounting for these known risks suggests smaller fetal brain volume is the most consistent predictor of lower toddler ND scores [3], emphasizing the importance of fetal brain dysmaturation for later outcomes.

Prenatal disturbances in brain growth have led to increasing focus on the role of the placenta for adverse neurodevelopmental outcomes in CHD [4]. The placenta is a complex organ that is integral to fetal growth and development, serves as the link between maternal and fetal health, and is important for both short- and long-term well-being of the fetus [5–8]. Current data suggest that placental maldevelopment and dysfunction, particularly vascular disturbances, occur with fetal CHD [9–15]. Placental vascular abnormalities are associated with neonatal neurologic conditions, such as hypoxic-ischemic encephalopathy and perinatal stroke in term-born infants, and with neurodevelopmental deficits in preterm-born infants [16–20]. While not directly focused on vascular changes, one study in infants with CHD reported higher rates of perioperative brain injury for those who had placental abnormalities [21]. Recently, data have suggested that the increasing severity of placental lesions correlates with smaller neonatal brain volumes in infants with severe CHD [22]. Collectively, these data support that placental vascular changes may be associated with brain dysmaturation and adverse neurodevelopmental outcomes in those with CHD.

The overlapping molecular pathways and parallel development of the placenta, heart, and brain support the possibility of shared genetic and epigenetic vulnerability across these organ systems [4, 23–28]. However, fetuses with CHD and genetic diagnoses are often excluded from magnetic resonance imaging (MRI) cohorts. The current study sought to investigate the association of placental vascular abnormalities, identified on clinical histopathology, with third-trimester total and regional brain volumes in fetuses with CHD with and without genetic diagnoses. We hypothesized that fetuses with a placental vascular abnormality would have smaller total and regional brain volumes, regardless of a genetic diagnosis.

## Methods

### Study design and participants

Data from two prospective, longitudinal fetal brain MRI studies, conducted at Boston Children’s Hospital (BCH) and St. Louis Children’s Hospital (SLCH), were retrospectively combined for the current study. Participants from BCH were enrolled from January 2014 to September 2023, and participants from SLCH were enrolled from June 2018 to April 2024. For both cohorts, pregnant women were eligible if they were 18–45 years of age and had a prenatal diagnosis of fetal moderate-severe CHD expected to require cardiac surgery in the first year of life [29]. Exclusion criteria were maternal CHD, MRI contraindication, multiple gestation pregnancy, fetal brain malformation, congenital infection, non-English speaking, or the clinician deemed recruitment was inappropriate (e.g., considering termination). For the BCH cohort, fetuses with known genetic syndromes or extracardiac congenital anomalies were excluded from prospective recruitment; however, those diagnosed after birth were included in the current study (with the exception of Trisomy 13, 18, and 21). For the SLCH cohort, fetuses with genetic diagnoses or extracardiac congenital anomalies (with the exception of Trisomy 13 or 18) were eligible for enrollment throughout the study period. For the SLCH cohort, non-English speaking was an exclusion criterion; however, it was not an exclusion for the BCH cohort. To be included in the current analysis, participants had to have at least one fetal brain MRI and clinical placental pathology data.

### Ethics approval and consent to participate

The study was approved by the local IRB at each center, participants signed an informed consent document before any study procedures were conducted, and the study was performed in accordance with the Declaration of Helsinki.

### Placental pathology data

Placental pathology data were obtained through maternal chart review and were categorized according to the Amsterdam Placental Workshop criteria [30], similar to our prior work [12]. A single rater reviewed all clinical pathology reports. Histopathologic vascular abnormalities were grouped as maternal vascular malperfusion (MVM), fetal vascular malperfusion (FVM), and delayed villous maturation (DVM). Briefly, injury to maternal vessels secondary to underperfusion or high-velocity malperfusion was categorized as MVM, lesions that occur due to fetal blood flow obstruction (such as cord occlusion or thromboses) were categorized as FVM, and maldevelopment of the vasculosyncytial membrane, the site of essential gaseous exchange in the placenta, was categorized as DVM [30]. Placental inflammation was categorized as acute or chronic. Acute inflammation included subchorionitis, chorionitis, chorioamnionitis, necrotizing chorioamnionitis, chorionic vasculitis, umbilical phlebitis, inflammation of the umbilical vein or artery, and necrotizing funistis; chronic inflammation included villitis of unknown etiology, chronic intervillositis, and chronic deciduitis [30].

### MRI acquisition and processing

Participants underwent up to two fetal MRIs during their pregnancy. For the BCH cohort, the first fetal MRI was targeted to occur between 18–30 weeks of gestation and the second between 36–40 weeks of gestation. For the SLCH cohort, fetal MRIs were performed at 28–30 and 34–36 weeks of gestation. The current study utilized data from the latest available MRI as the outcome to most accurately reflect the “ultimate” brain size of the fetus, acknowledging that brain development is still rapidly progressing even in the last few weeks of the pregnancy [31]. If a second MRI was not available, the first MRI was used. Because brain volumes change rapidly over gestation and the timing of MRI acquisition was broad, statistical analyses adjusted for gestational age effects, as described below.

MRIs were performed on a 3-Tesla Siemens scanner with similar acquisition parameters and post-processing methods across both study sites. Acquisition parameters have been previously published for both cohorts [32–34]. The BCH parameters included multi-planar repeated T2-weighted half Fourier acquisition single shot turbo spin echo (T2w-HASTE) sequences performed with a 2 or 4 interleaved acquisition; echo time 100 and 120 ms; repetition time of 1400–2000 ms; 2–3 mm slice thickness; no inter-slice gap; 256 × 204, 256 × 256, or 320 × 320 acquisition matrices; and in-plane resolution of 1 mm. Similarly, for SLCH, multi-planar repeated T2-wHASTE sequences were acquired with a 2 or 4 interleaved acquisition; echo time of 117 ms; repetition time of 1600 ms; 2–3 mm thickness slice; no inter-slice gap; 256 × 256 acquisition matrix; and in-plane resolution of 1 mm. Across both sites, a variable field of view was used based on maternal and fetal size.

Post-processing of the MRI data was completed using the same pipelines and software for both sites. The T2w-HASTE images were reconstructed using inter-slice motion and signal intensity non-uniformity corrections, the data were registered to a fetal brain MRI atlas common coordinate space, and an automatic atlas-based segmentation was applied followed by manual modification and extraction of volume measures [32, 35], as shown in supplementary Fig. 1. The relevant code for fetal brain segmentation can be found at https://github.com/IntelligentImaging/CRL-2025-MAS. The atlas, along with its labels, is available online at https://dataverse.harvard.edu/dataverse/CRL2025Atlas.

Regions of interest included cerebrospinal fluid (lateral ventricles, third and fourth ventricles, and extra axial fluid); developing white matter (subplate zone, intermediate zone, corpus callosum, internal capsule, hippocampal commissure, and fornix); fetal cortex (cortical plate and hippocampus); subcortical gray matter (caudate nucleus, lentiform nucleus, and amygdala); proliferative compartments (ventricular zone and ganglionic eminence); diencephalon (thalamus and subthalamic nucleus); brainstem; and cerebellum. Total brain volume was calculated as the sum of all parenchymal brain regions, and intracranial volume was calculated as the sum of total brain and cerebrospinal fluid volumes, as previously described [32, 36].

### MRI outcomes

Growing evidence supports the possibility of an association between structural and functional placental changes (including oxidative stress) with brain disturbances in CHD [37]. We hypothesized that placental vascular abnormalities would be associated with smaller developing white matter (a region particularly vulnerable to hypoxia/oxidative stress), which would also contribute to reduced total brain and increased cerebrospinal fluid volumes. Thus, total brain, cerebrospinal fluid, and developing white matter volumes were chosen as the primary outcome measures. The remaining seven brain regions were designated as secondary outcomes.

### Sociodemographic and clinical data

Maternal sociodemographic variables were obtained through self-report and included age, race, ethnicity, and maternal education. Maternal clinical and fetal variables were obtained from medical record review and direct interview. Maternal clinical data included tobacco, alcohol, or drug exposure during pregnancy, chronic or pregnancy-induced diabetic and hypertensive disorders (as defined by O’Hare et al. [12],), and presence of oligohydramnios or polyhydramnios. Fetal variables included fetal sex, cardiac diagnosis, genetic diagnosis or syndrome, associated congenital anomalies, intrauterine growth restriction defined as estimated fetal weight <10% at any prenatal ultrasound, gestational age at MRI and at birth, birth anthropometric measurements, and delivery mode. Birth anthropometric measurements were converted to gestational age z-scores using the World Health Organization growth standard for term infants and Olsen (BCH) or Fenton (SLCH) Growth Calculators for infants born before 37 weeks of gestation [38]. CHD diagnosis was determined from a fetal echocardiogram by a fetal cardiologist and then categorized as single or two-ventricle anatomy. The first postnatal echocardiogram was used to confirm prenatal diagnosis and ensure accurate categorization.

To evaluate the impact of genetics, fetuses with a genetic syndrome (i.e., VATER/VACTERL), pathogenic variant, or a significant associated congenital anomaly were defined as having a genetic abnormality. Molecular diagnoses were determined by prenatal and/or postnatal clinical genetic testing, including maternal free DNA testing, fluorescence in situ hybridization, karyotyping, chromosomal microarray analysis, exome sequencing, and/or targeted gene panels. Congenital anomalies (i.e., esophageal atresia and vertebral anomalies, consistent with VACTERL) were identified on prenatal or postnatal imaging.

### Statistical analysis

Due to varying gestational ages at MRI and known sex differences in fetal brain growth over pregnancy [39], residual brain volumes were calculated from all MRI data (including multiple scans) using generalized estimating equations with the identity link adjusting for fetal sex and a natural cubic spline of gestational age at MRI. This was done to normalize the outcome variables to fetal sex and gestational age and calculate residual brain volumes before model building. Then, univariable linear regression with generalized estimating equations was used to determine the association of placental vascular abnormality and genetic abnormality separately with residual brain volumes for all ten brain outcome measures. Multivariable regression models with generalized estimating equations were then used to jointly assess the association of placental vascular abnormality, genetic abnormality, and their potential interaction with residual brain volumes for all ten brain outcome measures, adjusting for maternal education and single ventricle anatomy as clinically relevant factors. Univariable analyses of study site, adverse maternal environment (diabetic disorders, hypertensive disorders, and tobacco, alcohol, or drug exposure), oligohydramnios or polyhydramnios, fetal sex, preterm birth, placental weight, and placental inflammation were also performed for consideration in the multivariable model. Generalized estimating equations were used throughout with an exchangeable correlation structure and identity link to account for potential non-normality or unequal variances in brain volumes across gestational ages. Histograms for residuals from all models were assessed for approximate normality with visual inspection. To provide a graphical representation of the group differences between no abnormality, placental abnormality only, genetic abnormality only, and placental plus genetic abnormality, an unadjusted analysis of variance (ANOVA) was performed. Multiple comparison correction was not used for analysis. All analyses were performed using R version 4.3.0 (R Foundation for Statistical Computing, Vienna, Austria).

## Results

### Cohort characteristics

Among 196 participants enrolled across the two centers during the study period (125 from BCH, 71 from SLCH), 121 had MRI and placental pathology data available for analysis (71 from BCH, 50 from SLCH) (Supplementary Fig. 2). Placental vascular abnormality was present in 46% (56/121) of participants, the most common of which was MVM (Table 1). With the exception of fetal genetic abnormality, maternal and fetal characteristics for those with *versus* without placental vascular abnormalities were similar, including placental weight and the frequency of placental inflammation (Table 1). The distribution of cardiac diagnoses by placental abnormality is displayed in Supplementary Table 1.

**Table 1:** Cohort characteristics.

	Any placental abnormality (*n* = 56)	No placental abnormality (*n* = 65)
Maternal characteristics		
Age at birth, years	30.7 ± 5.6	30.3 ± 5.7
Race		
Asian	2 (4)	0
Black	4 (7)	3 (5)
White	46 (82)	58 (89)
Other^a^	4 (7)	4 (6)
Hispanic ethnicity	3 (5)	3 (5)
Education (college or higher)	34 (61)	34 (52)
Tobacco, alcohol, or drug exposure	18 (32)	14 (22)
Diabetic disorder	11 (20)	9 (14)
Hypertensive disorder	6 (11)	5 (8)
Oligohydramnios/polyhydramnios	7 (12)	10 (15)
Fetal characteristics		
Male	27 (48)	38 (58)
Single ventricle anatomy	21 (38)	21 (32)
Genetic abnormality^b^	15 (27)	8 (12)
Intrauterine growth restriction	11 (20)	14 (23)
Gestational age at MRI, weeks	34 ± 3.7	34 ± 3.7
Gestational age at birth, weeks	38 ± 1.4	39 ± 1.3
Preterm birth (<37 weeks)	7 (12)	6 (9)
Birth weight, Z-score^c^	0.50 ± 0.96	0.68 ± 1.07
Birth head circumference, Z-score^c^	0.27 ± 1.13	0.17 ± 1.21
Delivery mode: emergent cesarean section	4 (9)	4 (7)
Placental characteristics		
Placental weight, grams	484 ± 119	490 ± 118
Placental inflammation	13 (23)	21 (32)
Acute	7 (12)	16 (25)
Chronic	7 (12)	9 (14)
Placental vascular abnormalities		
Maternal vascular malperfusion	39 (70)	0
Fetal vascular malperfusion	11 (20)	0
Delayed villous maturation	12 (21)	0
More than one placental abnormality^d^	6 (11)	0
Enrollment site		
Boston Children’s Hospital	27 (48)	44 (68)
St. Louis Children’s Hospital	29 (52)	21 (32)

Values are mean ± standard deviation or *n* (%).

^a^Other race in the any placental abnormality group includes American Indian/Alaska Native (*n* = 1), Native Hawaiian/Pacific Islander (*n* = 1), multiracial (*n* = 1), and unknown (*n* = 1). Other race in the no placental abnormality group includes Syrian and Italian (*n* = 1), Puerto Rican (*n* = 1), multiracial (*n* = 1), and unknown (*n* = 1).

^b^Fetuses with a genetic syndrome, pathogenic variant, or major associated congenital anomaly were defined as having a genetic abnormality.

^c^Birth measurements were converted to gestational age z-scores using the World Health Organization growth standard for term infants and Olsen (BCH) or Fenton (SLCH) Growth Calculators for infants born before 37 weeks gestation [38].

^d^Includes both maternal and fetal vascular malperfusion (*n* = 1), maternal vascular malperfusion and delayed villous maturation (*n* = 4), and fetal vascular malperfusion and delayed villous maturation (*n* = 1).

The frequency of genetic abnormality was slightly higher in those with *versus* without placental vascular abnormalities (27% *versus* 12%, Fisher’s exact *P* = 0.06). Those with genetic abnormalities had comparable birth weight, head circumference, frequency of intrauterine growth restriction, and placental weight to those without genetic abnormalities. The specific genetic abnormalities/congenital anomalies and their associated placental findings are shown in Table 2. The genetic testing performed in the cohort included maternal free DNA (*n* = 38), pre-implantation screening (*n* = 1), fluorescence in situ hybridization (*n* = 35), karyotyping (*n* = 37), chromosomal microarray analysis (*n* = 70), exome sequencing (*n* = 5), and targeted gene panels (*n* = 8).

**Table 2:** Genetic abnormalities and/or congenital anomalies with associated placental vascular findings.

Subject	Genetic abnormality	Placental vascular finding
1	2q13 deletion	None
2	5q duplication and 18q deletion	FVM
3	7q21.3q36.3 duplication	MVM
4	9q34.3 deletion^a^	FVM
5	Recurrent 17q12 duplication	MVM
6	22q11.2 deletion	DVM
7	22q11.2 deletion	FVM
8	22q11.2 deletion	MVM and DVM
9	22q11.2 deletion	None
10	Congenitally absent kidney^b^	MVM
11	*DNAH11* gene mutation	None
12	*DNAH11* gene mutation	None
13	Heterotaxy syndrome	MVM
14	Kabuki syndrome	FVM and DVM
15	*NOTCH1* gene mutation	None
16	Opitz G/BBB syndrome	MVM
17	Pathogenic variant in KDM5C^c^	DVM
18	Phelan-McDermid syndrome	None
19	Right cleft lip, submucous cleft palate, large congenital scalp nevus^d^	None
20	Spine segmentation abnormalities, ribs anomalies, abnormal stomach location^d^	MVM
21	Turner Syndrome	MVM and DVM
22	VATER/VACTERL	MVM
23	VATER/VACTERL	None

Molecular diagnoses were determined by prenatal and/or postnatal clinical genetic testing, including fluorescence in situ hybridization, karyotyping, chromosomal microarray, exome sequencing, and/or targeted gene panels. Congenital anomalies (i.e., esophageal atresia and vertebral anomalies, consistent with VACTERL) were identified on prenatal or postnatal imaging.

Participants shown here are all fetuses who met criteria for being defined as having genetic abnormalities. For the remaining cohort (*n* = 98), no genetic abnormality was identified on clinical genetic evaluation.

^a^Includes the entire *AGPAT2* gene and all the coding exons in the *NOTCH1* gene.

^b^Without molecular diagnosis.

^c^Pathogenic variants in this gene are associated with X-linked developmental disorder, Claes-Jensen type.

^d^Without molecular diagnosis, but geneticist reported high suspicion for underlying genetic etiology.

Supplementary Table 2 shows the cohort characteristics by study site. Compared to participants from Boston Children’s Hospital, participants from St. Louis Children’s Hospital had a younger maternal age (32.6 years *versus* 27.6 years, *P* < 0.001), were less likely to be college educated (68% *versus* 40%, *P* = 0.003), had a lower frequency of male fetuses (63% *versus* 40%, *P* = 0.02), were more likely to have a fetal genetic abnormality (8% *versus* 34%, *P* < 0.001), and had a higher frequency of DVM (3% *versus* 20%, *P* = 0.004).

### Univariable associations of placental vascular abnormality and genetic abnormality with fetal brain volumes

In univariable analysis, the presence of a placental vascular abnormality alone was not associated with smaller residual brain volumes for the primary (total brain, cerebrospinal fluid, developing white matter) or secondary (all other brain regions) outcomes (Table 3). When examining MVM, FVM, and DVM individually, there were also no associations with residual brain volumes (all *P* > 0.05).

**Table 3:** Univariable associations of placental vascular and genetic abnormalities with residual brain volumes (mL).

Region	Comparison between placental abnormality (*n* = 56) and no placental abnormality (*n* = 65)		Comparison between genetic abnormality^a^ (*n* = 23) and no genetic abnormality (*n* = 98)	
	Mean difference [95% CI]	*p*	Mean difference [95% CI]	*p*
Intracranial	–2.3 [–15.5, 10.9]	0.73	–9.7 [–29.1, 9.6]	0.32
Cerebrospinal fluid	–0.7 [–7.4, 6.0]	0.83	2.9 [–7.5, 13.3]	0.59
Total brain	–1.5 [–10.3, 7.3]	0.74	–12.5 [–24.0, –1.0]	0.03
Developing white matter	–0.1 [–5.3, 5.0]	0.96	–7.0 [–13.7, –0.4]	0.04
Fetal cortex	–1.0 [–4.5, 2.7]	0.60	–3.4 [–8.4, 1.6]	0.18
Subcortical gray matter	–0.01 [–0.2, 0.2]	0.96	–0.4 [–0.8, –0.1]	0.01
Proliferative compartments	0.1 [–0.3, 0.5]	0.64	–0.05 [–0.6, 0.5]	0.86
Diencephalon	–0.01 [–0.2, 0.2]	0.89	–0.3 [–0.6, –0.1]	0.02
Brainstem	–0.01 [–0.2, 0.2]	0.94	–0.4 [–0.6, –0.1]	0.002
Cerebellum	–0.4 [–1.0, 0.2]	0.16	–0.9 [–1.8, –0.002]	0.0496

^a^Fetuses with a genetic syndrome, pathogenic variant, or major associated congenital anomaly were defined as having a genetic abnormality.

Mean differences and confidence intervals (CI) are from unadjusted linear regressions of residual brain volumes using generalized estimating equations.

Also in univariable analysis, fetuses with genetic abnormalities had smaller residual total brain (–12.5 mL; 95% CI –24.0, –1.0) volume, as well as smaller residual volumes in the developing white matter, subcortical gray matter, diencephalon, brainstem, and cerebellum compared to those without genetic abnormalities (Table 3).

### Multivariable associations of placental vascular abnormality, genetic abnormality, and other factors on fetal brain volumes

Other than placental vascular and genetic abnormalities, after adjusting for single ventricle and maternal education, placental weight was also associated with fetal residual brain volumes. Its addition to multivariable models did not appreciably change the results; therefore, it was not included in the final models. None of the other risk factors considered, including placental inflammation, was predictive of residual brain volumes and thus was also not included in the final multivariable models. With respect to the study site, compared to fetuses from Boston Children’s Hospital, fetuses from St. Louis Children’s Hospital had smaller residual brain volumes for diencephalon (0.1 ± 0.5 *versus* –0.1 ± 0.6, *P* = 0.04) and brainstem (0.1 ± 0.4 *versus* –0.2 ± 0.5, *P* = 0.006) (Supplementary Table 2). Because the statistical approach already accounted for the factors that differed by site that may affect fetal brain volumes (i.e., maternal education, fetal genetic abnormalities, fetal sex), study site was not included in the final multivariable models.

**Fig. 1:**
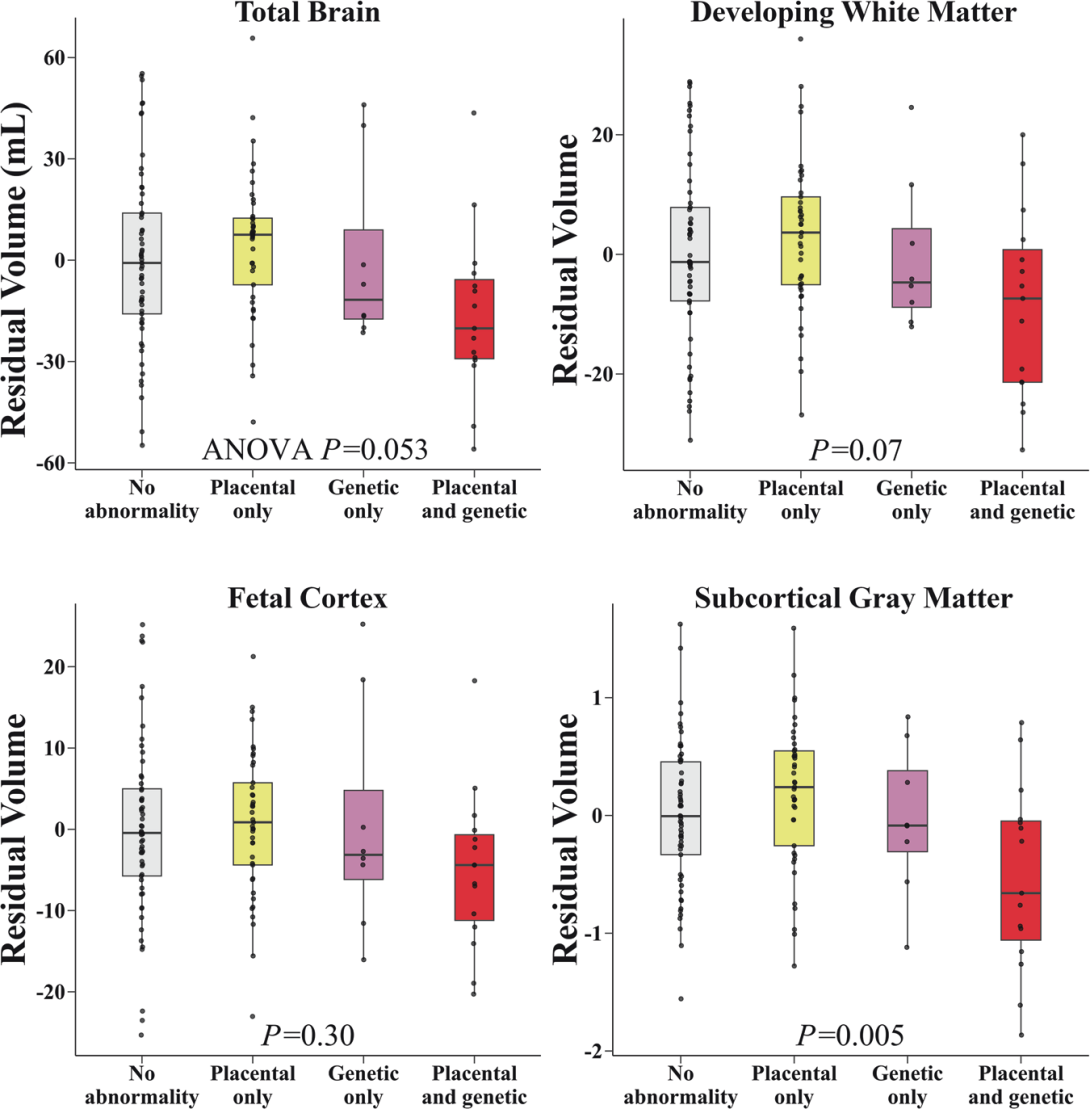
Graphical representation of the effect of placental vascular and genetic abnormalities. Boxplots comparing residual total and regional brain volumes (mL) after adjusting for fetal sex and natural cubic spline of gestational age at MRI for subjects with no placental or genetic abnormality (*n* = 57), placenta abnormality only (*n* = 41), genetic abnormality only (*n* = 8), and both placental and genetic abnormalities (*n* = 15). Analysis of variance (ANOVA) *P*-values comparing the four groups are reported. Across all regions shown, the group with both placental and genetic abnormalities had significantly lower residual volumes than those with no abnormality or placental abnormality only.

To delineate the joint effects of placental vascular and genetic abnormalities, models compared fetuses with placental vascular abnormality alone (without genetic abnormality), those with genetic abnormality alone (without placental vascular abnormality), and those with both placental vascular and genetic abnormalities *versus* fetuses with neither placental vascular nor genetic abnormalities. Selected brain regions across these four groups are presented in Fig. 1.

For the primary outcomes, fetuses with the combination of placental vascular and genetic abnormalities had smaller residual total brain volume compared to those without these abnormalities, adjusting for maternal education and single ventricle anatomy (Table 4). There was no difference in cerebrospinal fluid or developing white matter. For secondary outcomes, fetuses with the combination of placental vascular and genetic abnormalities had smaller residual brain volumes for subcortical gray matter, brainstem, and cerebellum (Table 4). Of note, there was a statistically significant interaction effect between placental vascular abnormality and genetic abnormality for residual intracranial and subcortical gray matter volumes (interaction *P* < 0.05 for each, Supplementary Table 3), with fetuses having both abnormalities being more severely impacted (Table 4). The interaction did not reach significance for cerebrospinal fluid, total brain, developing white matter, fetal cortex, proliferative compartments, diencephalon, brainstem, or cerebellum (Supplementary Table 3). Placental vascular abnormality alone and genetic abnormality alone were not independently associated with residual brain volumes in the multivariable models.

**Table 4:** Multivariable associations of placental vascular and genetic abnormalities with residual brain volumes (mL).

Region	Adjusted mean difference between placental abnormality only (*n* = 41) and no abnormality (*n* = 57)		Adjusted mean difference between genetic abnormality^a^ only (*n* = 8) and no abnormality (*n* = 57)			Adjusted mean difference between both placental and genetic abnormalities (*n* = 15) and no abnormality (*n* = 57)			*p*^b^
	Mean difference [95% CI]	*p*		Mean difference [95% CI]	*p*		Mean difference [95% CI]	*p*	
Intracranial	6.5 [–7.2, 20.2]	0.24		14.9 [–16.9, 46.6]	0.36		–19.1 [–41.7, 3.5]	0.10	0.12
Cerebrospinal fluid	2.3 [–4.5, 9.2]	0.50		13.4 [–4.3, 31.1]	0.14		–1.9 [–13.6, 9.7]	0.74	0.41
Total brain	4.2 [–5.1, 13.4]	0.38		1.2 [–17.3, 19.6]	0.90		–16.8 [–31.4, –2.3]	0.02	0.04
Developing white matter	3.0 [–2.4, 8.4]	0.27		0.7 [–7.9, 9.4]	0.87		–8.9 [–18.1, 0.3]	0.06	0.09
Fetal cortex	0.8 [–3.2, 4.7]	0.70		0.6 [–9.2, 10.4]	0.90		–5.3 [–10.8, 0.2]	0.06	0.18
Subcortical gray matter	0.2 [–0.1, 0.4]	0.16		0.02 [–0.4, 0.5]	0.93		–0.5 [–1.0, –0.08]	0.02	0.03
Proliferative compartments	0.02 [–0.4, 0.4]	0.94		–0.3 [–0.8, 0.2]	0.30		0.1 [–0.6, 0.8]	0.74	0.68
Diencephalon	0.1 [–0.1, 0.3]	0.28		–0.03 [–0.3, 0.2]	0.83		–0.4 [–0.8, 0.01]	0.06	0.11
Brainstem	0.1 [–0.1, 0.3]	0.28		–0.1 [–0.4, 0.1]	0.25		–0.4 [–0.7, –0.1]	0.01	0.02
Cerebellum	–0.04 [–0.6, 0.5]	0.90		–0.04 [–1.0, 0.9]	0.94		–1.4 [–2.7, –0.2]	0.02	0.16

Mean differences and confidence intervals (CI) compare each distinct group *versus* no placenta or genetic abnormality from linear regressions of residual brain volume using generalized estimating equations, adjusting for maternal education and single ventricle anatomy.

^a^Fetuses with a genetic syndrome, pathogenic variant, or major associated congenital anomaly were defined as having a genetic abnormality.

^b^3 Degrees of freedom.

## Discussion

This study investigated the association between placental vascular abnormalities and total and regional brain volumes in fetuses with moderate-severe CHD with and without genetic abnormalities, defined as a syndrome, molecular diagnosis, and/or associated congenital anomaly. Placental vascular abnormalities were common in the cohort, but when found in isolation, they were not associated with fetal brain volumes. However, the presence of a placental vascular abnormality was an important factor when the fetus also had a genetic abnormality. These results support the likelihood that complex intersecting pathways within the maternal-fetal environment relate to impaired fetal brain development in CHD.

Current data support high rates of placental disturbances in the CHD population [9–14]. Consistent with existing literature [9, 12], nearly half of our cohort had a placental vascular abnormality. By including fetuses with and without genetic abnormalities, we identified an important finding that the combination of placental and genetic abnormalities places fetuses with CHD at particularly heightened risk of smaller brain volumes. It is known that CHD patients with genetic abnormalities have poorer ND outcomes [1, 2], yet they are often excluded from neuroimaging studies, which limits understanding of the complex interplay of maternal and fetal risk factors contributing to altered brain development in CHD. In addition, excluding patients with genetic abnormalities makes findings less generalizable because of the high prevalence of genetic diagnoses within the CHD population [23, 40]. Only recently have investigators begun to expand MRI studies to include those with genetic disorders. For example, smaller regional brain volumes have been reported in children and adults with 22q11.2 deletion syndrome, Turner syndrome, and Noonan Syndrome [41, 42]. Data from the SLCH cohort were recently published, showing smaller placental and fetal brain volumes for fetuses with genetic abnormalities [33]. Findings from the current study (across two centers) of smaller total and regional brain volumes for CHD fetuses with genetic abnormalities add to the limited literature.

This study highlights a potential key role for genetics cutting across placental, heart, and brain health. Although placental abnormality alone did not relate to fetal brain volumes, it did become an important factor when a genetic abnormality was also present. Limited data suggest impaired syncytiotrophoblast formation and placental trophoblast inclusions are associated with genetic abnormalities [43, 44]. Damaging variants in chromatin-regulating genes, and Notch and Wnt signaling, are associated with the development of CHD and have been suggested to play a role in brain development and placental development [23–27]. Alterations in vasculogenesis and angiogenesis have also been implicated in placental, brain, and heart maldevelopment [45–47]. Thus, it is plausible that certain genetic diagnoses or pathogenic variants affect heart and placental development, creating a multiplicative effect of impaired cerebral oxygen and substrate delivery (from abnormal cardiac hemodynamics and placental dysfunction) that alters fetal brain growth. It is also possible that the genetic abnormality is causing heart, placental, and brain abnormalities irrespective of fetal/placental hemodynamics.

A notable finding of the current study was that there was no association of placental vascular abnormality alone with fetal brain volumes, or with MVM, FVM, or DVM individually. Placental vascular changes have been associated with adverse neurological outcomes for a wide spectrum of neonatal populations. For instance, FVM has been reported in 20% of neonates with hypoxic-ischemic encephalopathy compared to 7% of matched controls [19]. Placental thromboses, a finding consistent with FVM, are hypothesized to be the most likely etiology of perinatal arterial ischemic stroke [18, 20]. In the preterm population, placental abnormalities increase the risk for neurodevelopmental impairment [16, 17]. Specific to CHD, Nijman et al recently found an association between increased severity of placental abnormalities and smaller cerebral cortical and cerebellar volumes in neonates with severe CHD [22]. The lack of association in our cohort may relate to the timing of MRI, which was performed at a mean gestational age of 34 weeks (compared to a postmenstrual age of 39.5 weeks by Nijman and colleagues [22]). While this is only a 5–6-week difference between the cohorts, it does span a timeframe when rapid brain development is occurring [31, 48, 49], which could increase vulnerability of the cortex and cerebellum to placental insults at the end of gestation. Alternatively, the lack of association could relate to the desynchrony in timing of brain and placental assessments, the focus on placental vascular abnormalities, or that we did not capture lesion severity. Nijman et al. [22] applied a scoring system to evaluate a broad range of histopathologic abnormalities. While we did not find an association of placental inflammation with fetal brain volumes, it is still plausible that cumulative effects of other histopathologic abnormalities and/or lesion severity, which we were unable to fully capture, are important. The focus on vascular changes was intentional, given the high rates of vascular abnormalities in pregnancies with fetal CHD [9, 12, 15] that could alter placental function and thus affect fetal brain development. However, our results suggest that placental vascular changes may be less significant for the fetal brain unless cerebral maldevelopment is already present, as occurs with certain genetic diagnoses.

### Limitations

Several limitations exist in this study. First, there is the use of clinical placental pathology data, which led to substantial missing data that may have contributed to selection bias. Additionally, placental pathology reports generally only offer a qualitative, subjective description of gross and histologic pathology in the placenta. Although a single rater categorized findings using the standardized Amsterdam criteria [30], multiple unblinded pathologists at different institutions performed the examination of the placenta, which could have led to inconsistencies in reporting and may result in heterogeneity in the application of the Amsterdam definitions, as has been previously described [50]. Incorporation of quantitative metrics into clinical pathology may alleviate this challenge and enhance the utility of placental pathology for clinical practice. It is also possible that the chosen outcomes of MVM, FVM, and DVM do not fully capture the histopathologic processes affecting the fetal brain, as discussed above. Further, histopathology is not representative of factors such as placental function or efficiency and other tools, such as placental MRI, may lend further insight. Indeed, a recent publication of placental and fetal MRI identified smaller placental, fetal body, and fetal brain volumes but larger fetal to placental volume ratios for fetuses with CHD compared to controls [51]. These data suggest that placental adaptations in the setting of CHD may differ from other populations, such as fetal growth restriction, and underscore the importance of *in utero* assessment of development and function. A second challenge is that the sample size limited the power to detect effects of specific cardiac diagnoses and other risk factors, potential interactions between risk factors and gestational age, and potential interactions between placental and genetic abnormalities for analysis. Future, larger studies could address this issue, as well as the challenge of multiple comparisons in the current analysis. The need to group molecular genetic diagnoses, syndromes, and developmental defects together due to sample size also limits the generalizability of the findings, although only two fetuses in the cohort had a congenital anomaly without a molecular diagnosis. It is possible that those two fetuses had a different etiology for the anomaly, such as toxic exposure in utero, though this was not self-reported or evident by chart review. Ideally, genome sequencing would be sent on all patients to allow for the most comprehensive analysis. We were able to evaluate MVM, DVM, and FVM individually with our sample size, but there is still a possibility that we were underpowered, increasing the risk for a type II error. Lastly, site differences may have been underestimated despite a similar study design across the two centers. Indeed, our analyses show differences in cohort characteristics across the two sites. Notably, the frequency of genetic abnormalities varied by site, which reflects the one difference in study design across the centers—that patients with prenatally known genetic syndromes or congenital anomalies were excluded from the BCH cohort, whereas they were not for the SLCH cohort. This exclusion was universally applied to all eligible participants at BCH, but still could have contributed to selection bias. However, enrollment from two different sites did allow the inclusion of patients from distinct geographic regions, which lends important sociodemographic diversity (i.e., maternal age and education) for generalizability.

## Conclusions

The interactive effects of placental and genetic abnormalities on reduced brain volumes in fetuses with CHD support multifactorial prenatal pathways of disrupted brain development. Future studies integrating fetal neuroimaging, more comprehensive genetic testing such as genome sequencing, quantitative placental pathology evaluation, and methods of assessing placental function may improve understanding of the maternal-fetal mechanisms of altered brain development in CHD.

## Supplementary information

Supplementary Materials

## Data Availability

The datasets generated during and/or analysed during the current study are available from the corresponding author on reasonable request.
