# Supplementary material for: Brain volumes in fetuses with congenital heart disease and placental vascular abnormalities
Source: J Perinatol. 2026 Mar 13;46(7):1155–63. doi: 10.1038/s41372-026-02601-4 (PMC13267945; doi:10.1038/s41372-026-02601-4)

| Supplemental Table 1. Cardiac diagnosis by placental abnormality | | |
| --- | --- | --- |
|  | **Any placental abnormality (n=56)** | **No placental**  **abnormality**  **(n=65)** |
| Atrioventricular canal defect | 2 (4) | 1 (2) |
| Coarctation | 2 (4) | 4 (6) |
| Complex single ventricle | 7 (12) | 2 (3) |
| Double outlet right ventricle | 4 (7) | 1 (2) |
| Hypoplastic left heart syndrome | 10 (18) | 14 (2) |
| Interrupted aortic arch | 2 (4) | 0 (0) |
| Dextro-transposition of the great arteries | 10 (18) | 16 (25) |
| Tetralogy of Fallot | 9 (16) | 13 (20) |
| Tricuspid atresia | 1 (2) | 2 (3) |
| Truncus arteriosus | 2 (4) | 1 (2) |
| Other^a^ | 7 (12) | 11 (17) |

Values are n (%).

^a^Other cardiac diagnoses in the any placental abnormality group includes coarctation with multiple VSDs, bilateral SVC, and bicuspid aortic valve (n=1), Ebstein’s anomaly (n=1), fetal aortic stenosis with probable evolving hypoplastic left heart syndrome (n=1), levo-transposition of the great arteries (n=1), moderate-large ventricular septal defect (n=1), and pulmonary atresia (n=2). Other cardiac diagnoses in the no placental abnormality group includes critical pulmonary stenosis (n=1), dysplastic pulmonary valve with stenosis and regurgitation (n=1), fetal aortic stenosis with probable evolving hypoplastic left heart syndrome (n=2), large aortopulmonary window (n=1), levo-transposition of the great arteries (n=1), moderate-large ventricular septal defect (n=2), pulmonary atresia (n=1), Shone’s complex (n=1), and total anomalous pulmonary venous return (n=1).

| Supplemental Table 2. Cohort characteristics by Study Site | |  |
| --- | --- | --- |
|  | **Boston Children’s Hospital**  **(n=71)** | **St. Louis Children’s Hospital**  **(n=50)** |
| Maternal characteristics |  |  |
| Age at birth, years | 32.6 ± 4.6 | 27.6 ± 5.7 |
| Race |  |  |
| Asian | 2 (3) | 0 |
| Black | 2 (3) | 5 (10) |
| White | 63 (89) | 41 (82) |
| Other^a^ | 4 (6) | 4 (8) |
| Hispanic ethnicity | 5 (7) | 1 (2) |
| Education (college or higher) | 48 (68) | 20 (40) |
| Tobacco, alcohol, or drug exposure | 21 (30) | 11 (22) |
| Diabetic disorder | 10 (14) | 10 (20) |
| Hypertensive disorder | 6 (8) | 5 (10) |
| Oligohydramnios/polyhydramnios | 6 (8) | 11 (22) |
| Fetal characteristics |  |  |
| Male | 45 (63) | 20 (40) |
| Single ventricle anatomy | 24 (34) | 18 (36) |
| Cardiac diagnosis |  |  |
| AV Canal | 1 (1) | 2 (4) |
| Coarctation | 2 (3) | 4 (8) |
| Complex SV | 5 (7) | 4 (8) |
| DORV | 3 (4) | 2 (4) |
| HLHS | 13 (19) | 11 (22) |
| Interrupted aortic arch | 0 | 2 (4) |
| TGA | 18 (26) | 8 (16) |
| TOF | 13 (18) | 9 (18) |
| Tricuspid atresia | 3 (4) | 0 |
| Truncus arteriosus | 1 (1) | 2 (4) |
| Other^b^ | 12 (17) | 6 (12) |
| Genetic abnormality^c^ | 6 (8) | 17 (34) |
| Intrauterine growth restriction | 16 (23) | 9 (18) |
| Gestational age at MRI, weeks | 34 ± 4.4 | 35 ± 2.0 |
| Gestational age at birth, weeks | 39 ± 1.4 | 39 ± 1.3 |
| Preterm birth (<37 weeks) | 7 (10) | 6 (12) |
| Birth weight, Z-score^d^ | 0.40 ± 1.02 | 0.87 ± 0.96 |
| Birth head circumference, Z-score^d^ | 0.11 ± 1.10 | 0.40 ± 1.28 |
| Delivery mode: emergent cesarean section | 5 (7) | 4 (8) |
| Placental characteristics |  |  |
| Placental weight, grams | 481 ± 125 | 497 ± 107 |
| Placental inflammation | 23 (32) | 11 (22) |
| Acute | 18 (25) | 5 (10) |
| Chronic | 9 (13) | 7 (14) |
| Placental vascular abnormalities | 27 (38) | 29 (58) |
| Maternal vascular malperfusion | 19 (27) | 20 (40) |
| Fetal vascular malperfusion | 6 (8) | 5 (10) |
| Delayed villous maturation | 2 (3) | 10 (20) |
| More than one placental abnormality^e^ | 0 | 6 (12) |
| Residual brain volumes (mL) |  |  |
| Intracranial | 1.8 ± 36.7 | –1.4 ± 38.6 |
| Cerebrospinal fluid | 0.5 ± 17.0 | 0.7 ± 21.3 |
| Total brain | 1.1 ± 25.0 | –2.0 ± 24.9 |
| Developing white matter | 0.8 ± 14.8 | –1.1 ± 14.4 |
| Fetal cortex | –0.4 ± 10.0 | –0.2 ± 11.1 |
| Proliferative compartments | 0.1 ± 1.1 | –0.1 ± 1.1 |
| Subcortical gray matter | 0.1 ± 0.6 | –0.2 ± 0.7 |
| Diencephalon | 0.1 ± 0.5 | –0.1 ± 0.6 |
| Brainstem | 0.1 ± 0.4 | –0.2 ± 0.5 |
| Cerebellum | 0.2 ± 1.4 | –0.3 ± 1.9 |
| Values are mean ± standard deviation or n (%).  ^a^Other race in the Boston Children’s Hospital group includes Native Hawaiian/Pacific Islander (n=1), Syrian and Italian (n=1), Puerto Rican (n=1), and unknown (n=1). Other race in the St. Louis Children’s Hospital group includes American Indian/Alaska Native (n=1), multiracial (n=2), and unknown (n=1).  ^b^Other cardiac diagnoses in the Boston Children’s Hospital group includes coarctation with multiple VSDs, bilateral SVC, and bicuspid aortic valve (n=1), critical pulmonary stenosis (n=1), dysplastic pulmonary valve with stenosis and regurgitation (n=1), Ebstein’s anomaly (n=1), fetal aortic stenosis with probable evolving hypoplastic left heart syndrome (n=3), large aortopulmonary window (n=1), moderate-large ventricular septal defect (n=2), total anomalous pulmonary venous return (n=1), and Shone’s complex (n=1). Other cardiac diagnoses in the St. Louis Children’s Hospital group includes levo-transposition of the great arteries (n=2), moderate-large ventricular septal defect (n=1), pulmonary atresia (n=3).  ^c^Fetuses with a genetic syndrome, pathogenic variant, or major associated congenital anomaly were defined as having a genetic abnormality.  ^d^Birth measurements were converted to gestational age z-scores using the World Health Organization growth standard for term infants and Olson or Fenton Growth Calculator for infants born before 37 weeks gestation (34).  ^e^Includes both maternal and fetal vascular malperfusion (n=1), maternal vascular malperfusion and delayed villous maturation (n=4), and fetal vascular malperfusion and delayed villous maturation (n=1). | | |
|  | | |

| Supplemental Table 3. Associations of interactions between placental vascular and genetic abnormality with residual brain volumes (mL) | | |
| --- | --- | --- |
| **Region** | **Interaction** | |
|  | **β [95% CI]** | **p** |
| Intracranial | –40.5 [–79.0, –1.9] | 0.04 |
| Cerebrospinal fluid | –17.7 [–38.6, 3.2] | 0.10 |
| Total brain | –22.2 [–45.7, 1.4] | 0.07 |
| Developing white matter | –12.6 [–25.2, –0.1] | 0.05 |
| Fetal cortex | –6.7 [–18.0, 4.6] | 0.25 |
| Subcortical gray matter | –0.7 [–1.4, –0.1] | 0.02 |
| Proliferative compartments | 0.4 [–0.5, 1.3] | 0.41 |
| Diencephalon | –0.5 [–1.0, 0.004] | 0.05 |
| Brainstem | –0.4 [–0.8, 0.04] | 0.08 |
| Cerebellum | –1.3 [–2.9, 0.2] | 0.08 |
| Betas (β) and confidence intervals (CI) from linear regressions of residual brain volume using generalized estimating equations adjusting for maternal education and single ventricle anatomy. | | |

Supplemental Figure 1. Regional Brain Volume Segmentation. Reconstructed fetal brain MRI at 35.86 weeks in axial plane (A) with brain structures segmented (B). Red = Fetal cortex; Yellow = Developing white matter; Blue = Subcortical gray matter; Green = Proliferative compartments; Purple = Diencephalon; Orange = Brainstem; Light blue = Cerebrospinal fluid


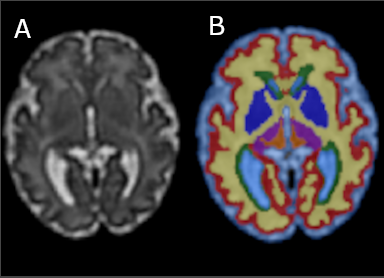


Supplemental Figure 2. Flow Diagram of Participant Inclusion and Exclusion by Study Site.

^a^Participant chose to terminate the pregnancy.

^b^Reasons for not undergoing MRI were preterm birth (n=2), inpatient admission on labor and delivery (n=1), claustrophobia/anxiety prohibiting completion of MRI (n=4), piercings that could not be removed (n=1), and inability to schedule before delivery (n=1).


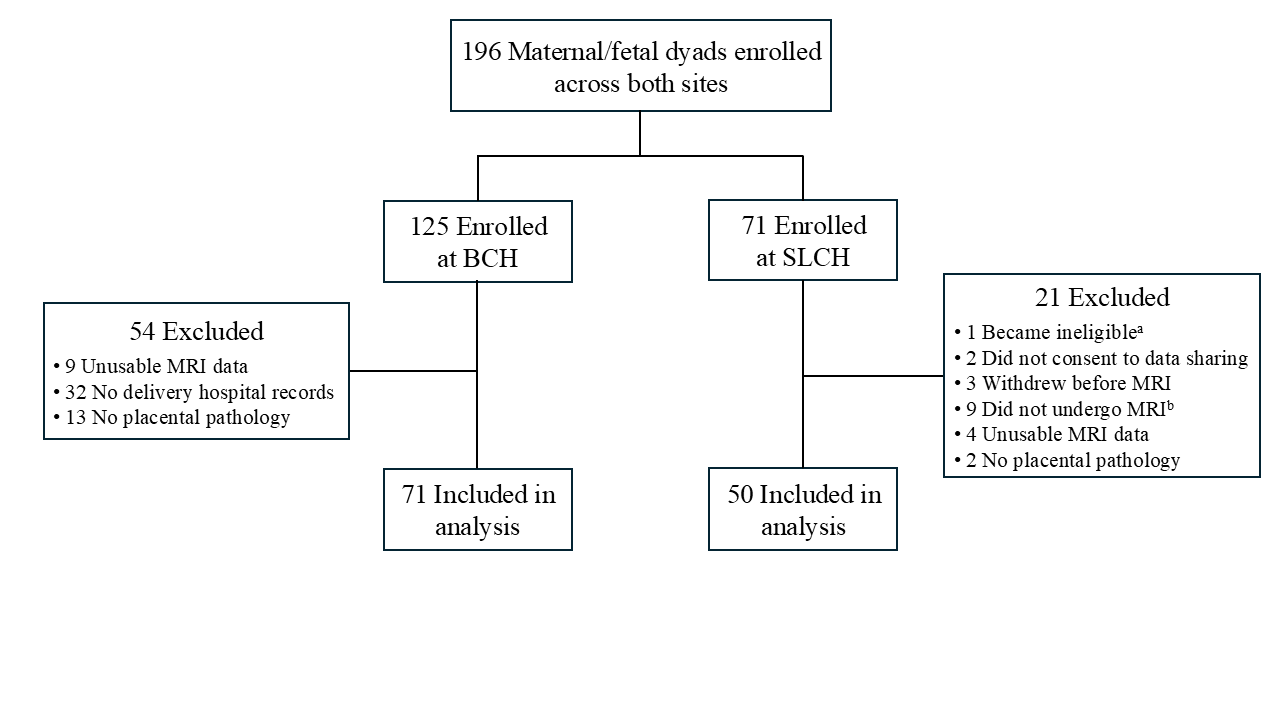

Supplement: Supplementary file 1 — Supplementary Materials [file 41372_2026_2601_MOESM1_ESM.docx]
